# Beyond the clinical eye: mapping intestinal parasitic infections and its risk factors among dogs and cats across Portugal

**DOI:** 10.3389/fvets.2026.1814054

**Published:** 2026-07-01

**Authors:** Bárbara Ferreira, Joana Ferrolho, Clarissa Faria, Olga Borges, Sérgio Sousa, Maria do Céu Sousa

**Affiliations:** 1 Faculty of Pharmacy, University of Coimbra, Coimbra, Portugal; 2 CIBB – Center for Innovative Biomedicine and Biotechnology, University of Coimbra, Coimbra, Portugal; 3 CIVG—Vasco da Gama Research Center, EUVG—Vasco da Gama University School, Coimbra, Portugal; 4 CNC-UC–Center for Neuroscience and Cell Biology, University of Coimbra, Coimbra, Portugal

**Keywords:** Ancylostomatidae, *Cryptosporidium*, *Cystoisospora*, *Giardia*, prevalence, *Toxocara*, zoonosis, one health

## Abstract

**Background:**

Intestinal parasitic infections in dogs and cats represent a significant clinical challenge and pose relevant zoonotic risks to public health. Epidemiological data on Portuguese companion animals remain scarce, with limited sample sizes and geographic coverage, leaving key gaps in understanding parasite prevalence and associated risk factors.

**Methods:**

This cross-sectional epidemiological study investigated the prevalence and associated risk factors of intestinal parasitism in 363 dogs and 345 cats, sampled from companion, breeding, shelter, and stray populations between November 2022 and April 2025. A total of 708 fecal samples were analyzed using coprological techniques (flotation and sedimentation), *Giardia* immunochromatographic test, and modified Ziehl-Neelsen staining.

**Results:**

Overall, 41.6% of dogs were positive for intestinal parasites, with protozoa and monoparasitism (61.2%) predominating. *Giardia lamblia* was the most frequently detected (44.7%), followed by Ancylostomatidae (16.5%), *Cystoisospora* spp. (15.4%), *Toxocara canis* (14.9%), *Trichuris vulpis* (4.3%), *Cryptosporidium* spp. (3.2%), and *Toxascaris leonina* (1%). Among cats, 42.6% tested positive, exhibiting greater parasite diversity, with a predominance of helminths and a higher occurrence of polyparasitism (116/211; 55%). The most prevalent parasite was *Toxocara cati* (30.3%), Ancylostomatidae (27.5%), *Cystoisospora* spp. (18%), *Aelurostrongylus abstrusus* (10.4%), *G. lamblia* (7.2%), and *Cryptosporidium* spp. (3.3%). Less frequent parasites included *Dipylidium caninum*, *Mesocestoides* spp., *Cystoisospora rivolta*, *Toxoplasma gondii*-like oocysts, and *Troglostrongylus*. Stray and shelter animals had significantly higher infection risks (OR = 2.3 for dogs; OR = 4.5 for cats). Age, abnormal fecal consistency, and seasonality were additional factors significantly associated with parasite positivity.

**Conclusion:**

Taken together, the detection of intestinal parasites in 41.6% of dogs and 42.6% of cats highlights a substantial burden, with parasites of zoonotic potential accounting for 73.9% of all positive results (295/399). These high rates point to critical gaps in preventive care among companion animal populations, underscoring the urgent need for strengthened surveillance, targeted treatment protocols, and enhanced public health education to reduce parasite burdens and mitigate zoonotic risks in Portugal.

## 1 Introduction

Domestic animals, including dogs and cats, are susceptible to a wide array of pathogenic agents, viruses, bacteria, fungi, and parasites, that can significantly impair their health and welfare (1, 2). Among these, parasitic diseases represent a pervasive and complex challenge due to their direct detrimental effects on animal health, causing a range of clinical manifestations, from mild discomfort to severe organ damage (3). Infectious diseases in domestic animals also play a crucial role in shaping human health outcomes, particularly through their potential to act as reservoirs of zoonotic pathogens (2).

Intestinal parasitic infections are among the most common health concerns affecting companion animals, particularly dogs and cats, with important implications for both animal welfare and public health (1). These infections are typically caused by a range of helminths and protozoa, including roundworms (*Toxocara* spp.), hookworms (*Ancylostoma* spp.), whipworms (*Trichuris* spp.), cestodes such as *Dipylidium caninum* and *Taenia* spp., and protozoan such as *Giardia lamblia, Cryptosporidium* spp., and *Cystoisospora* spp. Transmission often occurs through ingestion of infective stages from contaminated environments, predation, or vertical routes (such as transplacental or transmammary in puppies and kittens). Clinical manifestations can vary from subclinical infections to gastrointestinal signs such as diarrhea, vomiting, weight loss, and poor growth, particularly in young or immunocompromised animals. Beyond their veterinary significance, several of these parasites have zoonotic potential, posing risks to human health, especially in children and immunosuppressed individuals (1). For example, giardiosis and cryptosporidiosis are among the most prevalent enteric protozoa infections, causing significant gastrointestinal illness characterized by diarrhea, abdominal cramps, malabsorption, and dehydration (4, 5). Other infections such as toxocariosis pose significant zoonotic risks through environmental contamination with embryonated eggs or larvae, potentially leading to visceral, ocular, or neural larva migrans in humans (6, 7, 8).

Understanding the dynamics of infection in domestic animal populations is therefore essential for early detection, prevention, and control of diseases that threaten both animal and human health (9). Consequently, routine fecal screening, strategic deworming protocols, and proper hygiene practices are essential components of parasite control (3). Coproparasitological diagnostic methods continue to be the cornerstone for detecting intestinal parasitism in companion animals (3, 10).

Challenges in anthelmintic treatment arise from factors such as inappropriate drug choice, incorrect dosing, pharmacokinetic variability, emerging drug resistance, and persistent environmental exposure leading to rapid reinfection (11, 12).

The prevalence and diversity of parasitic infections varies according to geographic and climatic conditions, the animal’s lifestyle (stray *versus* pets), deworming practices, and broader environmental factors. In Europe, *Toxocara* spp. are among the most frequently reported nematodes, detected in up to 20% of pet dogs and cats (13–17). Particularly for *T. cati*, a pooled prevalence of 17.8% is reported for cats in Europe, aligning with the worldwide prevalence (17%) (18). Other commonly detected parasites include hookworms of the family Ancylostomatidae, with reported rates of up to 27% (14, 15, 19–23) and whipworms (*Trichuris vulpis*) (20, 21, 24). Protozoa including *Giardia lamblia*, *Cryptosporidium* spp., and *Cystoisospora* spp. have also been detected in domestic animals. *Giardia lamblia* as the main parasite encountered in dogs (22, 25–27) and *Cystoisospora* spp. in cats (13, 28–30).

Portugal exhibits predominantly temperate Mediterranean environmental conditions characterized by mild, rainy winters and warm to hot, dry summers (31), which play a key role in the dissemination of parasites affecting companion animals. The higher humidity and moderate temperatures during autumn and winter favor the survival, embryonation, and persistence of many intestinal parasite stages in the environment, such as eggs and larvae of helminths and cysts of protozoa (7, 32, 8). These conditions enhance environmental contamination in public spaces, kennels, and household settings, increasing the likelihood of transmission. Conversely, the hot and dry summer period can reduce the viability of some parasites due to desiccation; however, several species possess resistant forms that allow them to persist even under harsh conditions, maintaining a baseline risk of infection year-round. Overall, these environmental factors, combined with heterogeneous standards of pet care, a substantial stray animal population (33) and insufficient of deworming practices (34) creates favorable conditions for the transmission of gastrointestinal parasites. Epidemiological data on intestinal parasitic infections in companion animals in Portugal remain limited, often based on small sample sizes and restricted geographic coverage, thereby leaving important gaps in the understanding of parasite prevalence and associated risk factors.

This study aims to provide an up-to-date overview of intestinal parasites in dogs and cats across the country, assessing prevalence, diversity, and associated risk factors. By elucidating epidemiological patterns and zoonotic implications, it seeks to support enhanced surveillance, targeted control programs, and public health initiatives to mitigate the impact of these parasites on animal and human populations.

## 2 Materials and methods

### 2.1 Study design

**Figure 1:**
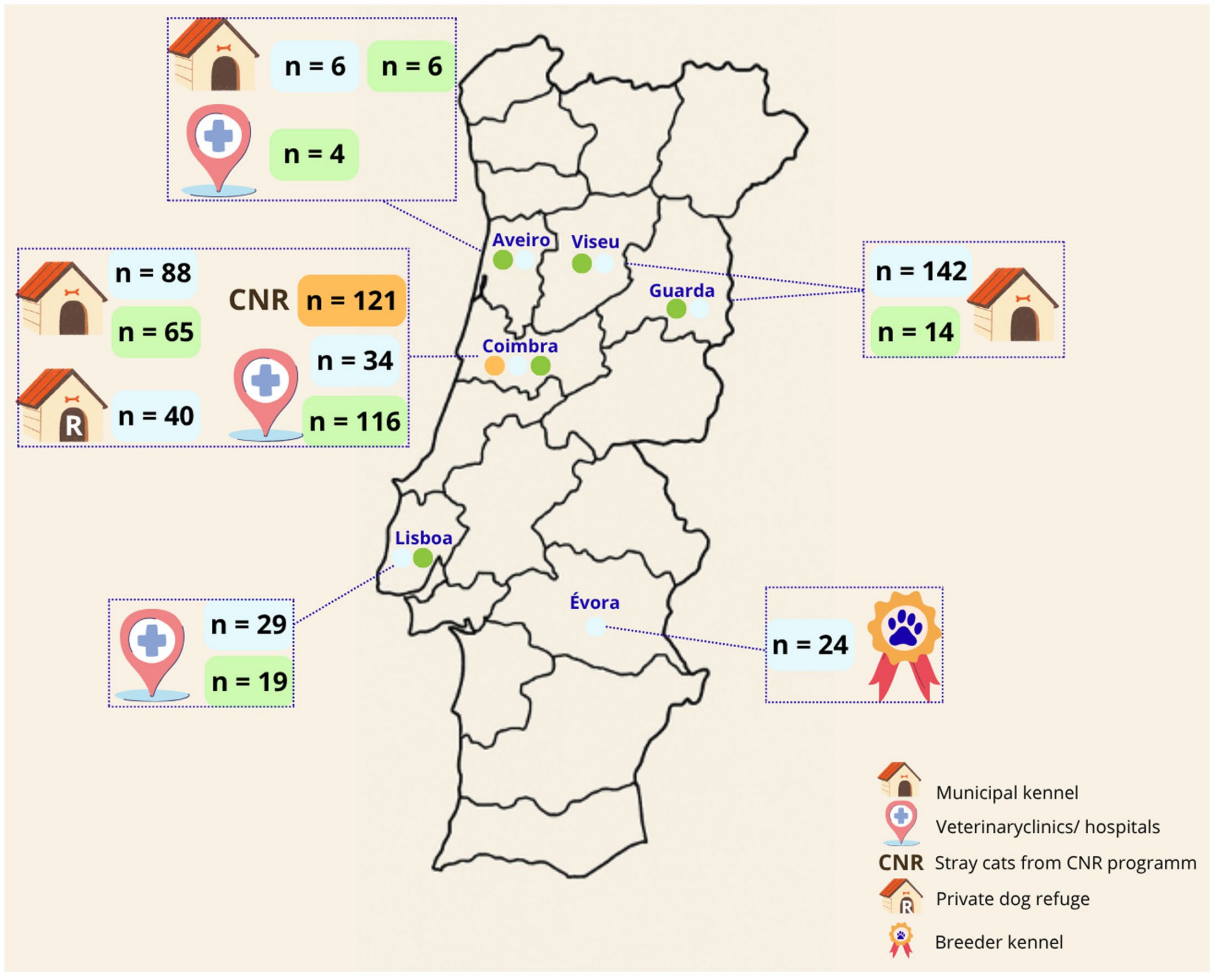
Geographic distribution of the fecal samples collected from dogs and cats across districts in Portugal. Blue boxes refer to samples from dogs, whereas green and orange boxes correspond to samples from cats. The figure was created by the authors using Canva design elements and an AI-generated base map.

This cross-sectional study was carried out from November 2022 to April 2025 to provide a representative characterization of intestinal parasites present in companion animals across Portugal. A total of 708 fecal samples were examined, comprising 363 from dogs and 345 from cats. These samples were collected from diverse sources including veterinary clinics and hospitals, municipal shelters, private kennels, and free-roaming cats enrolled in the Capture-Neuter-Return (CNR) program. The samples represented six distinct districts throughout Portugal (Aveiro, Viseu, Guarda, Coimbra, Lisboa and Évora) (Figure 1). The six districts were selected primarily based on the cooperation of local institutions and their readiness to provide samples, and secondly due to include different territorial profiles. District of Lisboa represents a major metropolitan area; Aveiro and Coimbra reflect semi urban coastal contexts; Viseu and Guarda capture interior and predominantly rural areas in the north central region; and Évora adds representation from the southern Alentejo. Information on climatic variables across the different districts and throughout the sample collection period is reported in the Supplementary Tables S1–S4. Animals were selected via convenience sampling from three primary groups: (i) pets presented for veterinary consultation at participating clinics and hospitals; (ii) animals temporarily housed within municipal shelters or private kennels; and (iii) free-roaming cats enrolled in the CNR program. Inclusion in the study depended on the accessibility and willingness of the animals, with consent obtained from owners for privately-owned animals and institutional approval for those housed in shelters.

### 2.2 Sample collection and data recording

Fecal samples were collected immediately after defecation using sterile, pre-labeled containers and transported under refrigeration at 4 °C to the Parasitology Laboratory of the University School Vasco da Gama on the same day as collection. For samples obtained from municipal shelters, collection was carried out in the morning following routine cleaning of animal enclosures to ensure the freshness of specimens. All samples were stored under refrigeration until processing, which was performed within 48 h of arrival at the laboratory. For each fecal sample, comprehensive metadata were systematically recorded to support thorough analysis and ensure reproducibility: (i) Species (dog or cat); (ii) Origin, categorized as follows: Veterinary clinics and hospitals (privately owned dogs and cats); Breeder kennels (breeder-owned dogs); Municipal shelters (stray dogs and cats); Private refuges (stray dogs and cats); Controlled colonies under veterinary supervision within the CNR program (stray cats); (iii) Age group, defined as: Puppy/kitten (<6 months); Young (6–11 months); Adult (1–10 years); Senior (>10 years); (iv) Sex (female or male); (v) Breed; (vi) History of antiparasitic treatment within the previous 3 months (when available).

### 2.3 Coprological analysis

#### 2.3.1 Macroscopic examination

All fecal samples were initially subjected to a macroscopic examination to detect visible parasitic elements, such as adult roundworms and cestode proglottids. Fecal consistency was assessed and recorded using the Purina Fecal Scoring System (Nestlé Purina, St. Louis, USA), categorized as follows: (i) Score 1 indicate hard, dry pellets, often expelled with difficulty and leaving little or no residue when picked up; (ii) Score 2–3 indicate normal consistency; (iii) Score 4–5 denotes soft but formed feces, with a moist texture and distinct shape that may pile rather than form distinct logs; (iv) Score 6 correspond to very soft to liquid, unformed stools, and (v) score 7 representing watery diarrhea. This scoring system provides a standardized and practical method to quantify fecal consistency, facilitating clinical assessment of gastrointestinal health and monitoring of disease progression or resolution.

#### 2.3.2 Flotation technique

Zinc sulfate flotation, a concentration technique, was performed following Zajac and Conboy (10) with slight modifications. Briefly, 2 g of feces were weighed and thoroughly mixed with 7 mL of zinc sulfate solution (specific gravity 1.18) in a plastic cup. The suspension was filtered through a metal strainer to remove coarse debris and transferred to a glass centrifuge tube. Additional zinc sulfate solution was added until a convex meniscus formed atop the tube, upon which a coverslip was carefully placed. After a 10-min incubation, the coverslip was gently removed, mounted on a glass microscope slide, and examined under an Olympus CH20 microscope (Olympus Corporation, Tokyo, Japan) at magnifications ranging from 100x to 400x. Parasitic elements, including eggs, cysts, oocysts, or larvae, were identified to genus or species level based on morphological criteria (10).

#### 2.3.3 Formalin–ethyl acetate sedimentation method

To enhance parasite detection, all samples were additionally processed using the formalin–ethyl acetate sedimentation technique (10, 35). Briefly, 2 g of feces were homogenized with 10% formalin, then strained through a fine mesh sieve and transferred into a 15 mL conical centrifuge tube. Subsequently, 3 mL of ethyl acetate were added. The tube was vigorously shaken to emulsify the mixture and centrifuged at 2500 rpm for 10 min (Sigma 3 K15, Harz, Germany). After centrifugation, supernatants were carefully decanted, and a drop of sediment was microscopically examined following the same procedures used in zinc sulfate flotation. Remaining sediment was stored at 4 °C pending further analysis via modified Ziehl–Neelsen staining.

#### 2.3.4 Immunochromatographic detection of *Giardia*

A commercial coproantigen immunochromatographic test (Giardia+ MonlabTest®, Monlab, Barcelona, Spain) was used to detect *Giardia* antigens (CWP1 and/or α_1_-giardin) according to manufacturer instructions. Approximately 150 mg of feces were sampled with a collection stick, targeting at least three distinct areas. The stick was returned to the buffer vial, closed, and shaken vigorously to homogenize. Four drops of the processed sample were then applied to the test device’s sample well. Results were read within 10 min: the presence of both a red test line and green control line indicated positivity; a single green control line denoted a negative result; tests lacking the control line were considered invalid and repeated.

#### 2.3.5 Modified Ziehl–Neelsen staining technique

*Cryptosporidium* spp. oocysts were detected using a modified Ziehl–Neelsen stain adapted from Henriksen and Pohlenz (36). Thin smears were prepared by pipetting 10 μL of formalin-fixed fecal sediment onto glass slides, air-dried at room temperature, and fixed with absolute methanol for 5 min. Slides were flooded with 1% phenicated fuchsin (Sigma-Aldrich, Darmstadt, Germany) for 15 min, rinsed with distilled water, decolorized using 10% acid–alcohol for 1 min, rinsed again, and counterstained with 3% malachite green (Certistain, Merck, Darmstadt, Germany) for 5 min. After final rinsing and air-drying, slides were microscopically examined under an Olympus CH20 (Olympus Corporation, Tokyo, Japan) at 400x and 1,000x magnification. *Cryptosporidium* oocysts appeared as bright red, spherical to ovoid structures against a blue/green background. A minimum of 100 microscopic fields per slide were assessed before declaring samples negative.

### 2.4 Data management and statistical analysis

All relevant variables related to fecal samples, including sample identification, animal metadata, and parasitological findings, were systematically entered and organized using Microsoft Excel® (Microsoft Corporation, Redmond, USA; version 16.43, 2019). Statistical analyses were conducted using GraphPad Prism version 6.0d (GraphPad Software, San Diego, USA). Categorical variables were summarized as frequencies and percentages. Associations between categorical variables were evaluated by Pearson’s chi-squared test or Fisher’s exact test, as appropriate. Statistical significance was set at a *p*-value of < 0.05. Additionally, odds ratios (OR), together with corresponding 95% confidence intervals (CI), were calculated to estimate the strength and direction of associations.

## 3 Results

Between November 2022 and April 2025, a total of 363 and 345 fecal samples from dogs and cats, respectively, were analyzed for the presence of endoparasites. Fecal examinations revealed that 151 dogs (41.6%) and 147 cats (42.6%) tested positive for one or more parasites over the 4 years, while 212 dogs (58.4%) and 198 cats (57.4%) tested negative (Table 1).

**Table 1:** Overall prevalence of intestinal parasites in dogs and cats.

Year of collection	Dogs (*n* = 363)			Cats (*n* = 345)		
	No. positive (%)	No. negative (%)	Total	No. positive (%)	No. negative (%)	Total
2022	5 (1.4)	7 (1.9)	12 (3.3)	19 (5.5)	11 (3.2)	30 (8.7)
2023	58 (16)	83 (22.9)	141 (38.9)	68 (19.7)	77 (22.3)	145 (42)
2024	85 (23.4)	118 (32.5)	203 (55.9)	60 (17.4)	106 (30.7)	166 (48.1)
2025	3 (0.8)	4 (1.1)	7 (1.9)	0 (0)	4 (1.2)	4 (1.2)
Total	**151 (41.6)**	**212 (58.4)**	**363 (100)**	**147 (42.6)**	**198 (57.4)**	**345 (100)**

Regarding the dog samples, the characteristics of animals testing positive and negative for intestinal parasites are summarized in Table 2. A total of 363 fecal samples were categorized into four groups based on their origin: veterinary clinics and hospitals (pet dogs; *n* = 63, 17.3%), breeder kennels (breeder dogs; *n* = 24, 6.6%), municipal kennels (stray dogs; *n* = 236, 65.1%), and private dog refuges (stray dogs; *n* = 40, 11%).

**Table 2:** Demographic and origin characteristics of dogs according to intestinal parasite status.

**Variable**	**No. (%) positive samples**	**No. (%) negative samples**	**Total**
Origin			
Veterinary clinics/hospitals (Pet dogs)	15 (4.1)	48 (13.2)	63 (17.3)
District of Coimbra	7 (1.9)	27 (7.4)	34 (9.3)
District of Lisboa	8 (2.2)	21 (5.8)	29 (8)
Breeder kennel (Breeder dogs)			
District of Évora	21 (5.8)	3 (0.8)	24 (6.6)
Municipal kennels (Stray dogs)	91 (25.1)	145 (39.9)	236 (65.1)
District of Aveiro	4 (1.1)	2 (0.6)	6 (1. 7)
District of Coimbra	38 (10.5)	50 (13.8)	88 (24.3)
Districts of Guarda/ Viseu	49 (13.5)	93 (25.6)	142 (39.1)
Private dog refuge (Stray dogs)			
District of Coimbra	24 (6.6)	16 (4.4)	40 (11)
Age group			
<6 months	50 (13.8)	18 (5)	68 (18.7)
6–11 months	2 (0.6)	6 (1.7)	8 (2.2)
1–10 years	98 (27)	183 (50.4)	281 (77.4)
>10 years	1 (0.3)	5 (1.4)	6 (1.7)
Sex			
Female	73 (20.1)	112 (30.9)	185 (51)
Male	78 (21.5)	100 (27.5)	178 (49)
Breed			
Mixed breed	122 (33.6)	185 (51)	307 (84.6)
Purebred	29 (8)	27 (7.4)	56 (15.4)
Season of sample collection			
Autumn	30 (8.3)	26 (7.2)	56 (15.4)
Winter	96 (26.5)	159 (43.8)	255 (70.2)
Spring	23 (6.3)	23 (6.3)	46 (12.7)
Summer	2 (10.5)	4 (1.1)	6 (1.7)
Diarrhea			
With	42 (11.6)	24 (6.6)	66 (18.2)
Without	109 (30)	188 (51.8)	297 (81.8)

Most samples were obtained from municipal kennels across several districts in Portugal, namely Guarda/Viseu (*n* = 142, 39.1%), Coimbra (*n* = 88, 24.3%), and Aveiro (*n* = 6, 1.7%). Dogs aged 1–10 years were the predominant group (*n* = 281, 77.4%).

The sex distribution was approximately equal, with females comprising 51% and males 49% of the sample population.

Mixed breed dogs represented the majority of the population (122 out of 307; 84.6%), while purebreds accounted for 15.4%. Nineteen distinct breeds were identified, with the Weimaraner (*n* = 18) being the most prevalent, followed by the Border Collie (*n* = 7) and the French Bulldog (*n* = 7) (Supplementary Table S5). Seasonal sample collection was predominantly during autumn (15.4%) and winter (70.2%) (Table 2).

Table 3 displays the frequency of each endoparasite genus identified in the dog population. The most frequently detected parasite was *G. lamblia* (44.7%), followed by Ancylostomatidae (16.5%), *Cystoisospora* canis (15.4%), *Toxocara canis* (14.9%), *Trichuris vulpis* (4.3%), *Cryptosporidium* spp. (3.2%), and *Toxascaris leonina* (1%). Overall, seven distinct species were identified, with protozoa constituting the most prevalent parasite group (63.3%).

**Table 3:** Prevalence of intestinal protozoa and helminths species in dogs, with distribution of mono- and polyparasitism.

Parasites	No (%) (*n* = 188)	Monoparasitism No (%) (*n* = 115)	Polyparasitism No (%) (*n* = 73)
Helminths			
Ancylostomatidae*	31 (16.5)	16 (14)	15 (21)
Toxocara canis*	28 (14.9)	19 (16.5)	9 (12)
*Trichuris vulpis.*	8 (4.3)	2 (1.7)	6 (8)
*Toxascaris leonina*	2 (1.0)	0 (0)	2 (3)
Total of helminths species	69 (36.7)	37 (32.2)	32 (44)
Protozoa			
Giardia lamblia*	84 (44.7)	60 (52)	24 (33)
*Cystoisospora canis*	29 (15.4)	15 (13)	14 (19)
*Cryptosporidium* spp.*	6 (3.2)	3 (3)	3 (4)
Total of protozoa species	119 (63.3)	78 (68)	41 (56)

* Zoonotic species.

Among dogs, parasitic infection was predominantly characterized by the presence of a single parasite species, indicative of monoparasitism, which accounted for 61.2% (115/188) of infections.

A deeper examination of the dog samples’ origin revealed that parasitic prevalence was highest among breeder dogs, with 21 out of 24 samples (87.5%) testing positive (Table 4). Of these, 21 animals were infected with a single parasite species, *G. lamblia*. It should be noted that all samples were obtained from a single private breeding kennel located in Évora, in Alentejo region of Portugal. The facility houses Weimaraner and Border Collie dogs, and sampling was conducted during a single time point, encompassing puppies younger than 6 months of age and their mothers, covering litters of both breeds. Therefore, this result is specific to that facility and may reflect a broader clustering bias among breeder dogs.

**Table 4:** Association between dog characteristics and prevalence of intestinal parasites: prevalence, odds ratios, and statistical significance.

Variable	Positive/total samples	Prevalence (%)	Odds ratio [95% CI]	*p-*value
Origin				
Veterinary clinics/hospitals (Pet dogs)	15/63	23.8	0.4 [0.1966–0.7098]	0.0018**
Breeder kennel (Breeder dogs)	21/24	87.5	11.3 [3.379–36.19]	<0.0001****
Municipal kennels (Stray dogs)	91/236	38.6		0.1188 *ns*
Private dog refuge (Stray dogs)	24/40	60	2.3 [1.167–4.431]	0.0166*
Age group				
<6 months	50/68	73.5	5.3 [2.974–9.646]	<0.0001****
6–11 months	2/8	25		0.4772
1–10 years	98/281	34.9	0.3 [0.1760–0.4830]	<0.0001****
>10 years	1/6	16.7		0.4072
Diarrhea				
With	42/66	63.6	3.0 [1.1760–5.258]	<0.0001****
Without	109/297	36.7		
Season				
Autumn	30/56	53.6		0.0556
Winter	96/255	37.6	0.6 [0.3668–0.9189]	0.0203*
Spring	23/46	50		0.2626
Summer	2/6	33.3		>0.999

CI: confidence interval. *p*-values: *, **, ***, **** significant difference at *p* < 0.05, *p* < 0.01, *p* < 0.001 and *p* < 0.0001, respectively.

Among the different canine groups assessed, pet dogs exhibited the lowest susceptibility to parasitic infection (odds ratio [OR] = 0.4; 95% confidence interval [CI] 0.197–0.710; *p* = 0.0018), whereas stray dogs had a 2.3-fold increased risk and breeder dogs an 11.3-fold increased risk of infection (Table 4).

Using pet dogs as the reference group, breeder dogs had a 22.4-fold higher risk (*p* < 0.0001), stray dogs from private refuge shelters had a 4.8-fold higher risk (*p* = 0.0003), and stray dogs from municipal kennels showed a 2-fold higher risk (*p* = 0.0374) of harboring intestinal parasites (Table 5).

**Table 5:** Association between dog origin and the prevalence of intestinal parasites: odds ratios and statistical significance.

Variable – Origin	Positive/total samples	Prevalence (%)	Odds ratio [95% CI]	*p-*value
Veterinary clinics/hospitals (Pet dogs)	15/63	23.8	§	
Breeder kennel (Breeder dogs)	21/24	87.5	22.4 [6.354–75.55]	<0.0001***
Municipal kennels (Stray dogs)	91/236	38.6	2 [0.0132–0.1574]	0.0374*
Private dog refuge (Stray dogs)	24/40	60	4.8 [2.076–10.77]	0.0003***

CI: confidence interval. §: Reference group. *p*-values: *, ***, significant difference at *p* < 0.05 and *p* < 0.001, respectively.

The highest prevalence of parasitism was observed in puppies under 6 months of age, who also demonstrated the greatest risk compared to older age groups (odds ratio [OR] = 5.3; 95% confidence interval [CI]: 2.974–9.646; *p* < 0.0001) (Table 4).

Another significant risk factor identified was abnormal fecal consistency, specifically diarrhea (scores 6 and 7, Purina Fecal Scoring System), which correlated strongly with intestinal parasite positivity (OR = 3.0; 95% CI: 1.176–5.258; *p* < 0.0001) (Table 4).

Seasonal variation was also evident, with the highest overall parasite prevalence recorded in autumn (53.6%) and spring (50%), seasons characterized by moderate temperatures and variable precipitation (Table 4). Winter showed a moderate prevalence (37.6%), despite having the highest absolute number of cases. The lowest prevalence was recorded in Summer (33.3%). It should be noted that the summer sample size was very small (*n* = 6), which may limit the reliability and stability of the prevalence estimate for this season.

Due to its distinctive characteristics and the uniform parasite profile observed, the breeder dog group was excluded from the parasite diversity analysis to avoid biasing the overall results. Bivariate analysis revealed a significant association between helminth positivity and stray dog status, with stray dogs exhibiting a 3.8-fold increased risk of helminth infection (odds ratio [OR] = 3.8; 95% confidence interval [CI], 1.373–10.01; *p* = 0.0095) (Table 6).

**Table 6:** Statistical analysis of intestinal parasite diversity in dogs (excluding breeder dogs): associations with host characteristics and parasite prevalence.

Variable	Category	Helminths			Protozoa			Monoparasitism			Polyparasitism		
		No. (%)	Odds ratio [95% CI]	*p-*value	*n* (%)	Odds ratio [95% CI]	*p-*value	*n* (%)	Odds ratio [95% CI]	*p-*value	*n* (%)	Odds ratio [95% CI]	*p-*value
Origin	Stray dogs (56/276)	56 (16.5)	3.8 [1.373–10.01]	0.0095**	74 (21.8)		0.3419	82 (24.2)		0.1180	33 (9.7)		0.1138
	Pet dogs (4/63)	4 (1.2)			13 (3.8)			12 (3.5)			3 (0.9)		
Age group	<6 months (30/48)	7 (2.1)		0.6840	26 (7.7)	4.5 [2.378–8.554]	<0.0001****	25 (7.4)	3.5 [1.880–6.578]	0.0001***	5 (1.5)		>0.9999 *ns*
	6–11 months (1/7)	1 (0.3)		>0.9999	1 (0.3)		0.6826	0 (0)		0.1968	1 (0.3)		0.5477 *ns*
	1–10 years (98/278)	51 (15)		0.5821	60 (17.7)	0.35 [0.1966–0.6237]	0.0006***	68 (20)	*0.44* [0.2502–0.7822]	0.0068**	30 (8.8)		>0.9999
	>10 years (1/6)	1 (0.3)		>0.9999	0 (0)		0.3445	1 (0.3)		>0.9999	0 (0)		>0.9999
Diarrhea	With (35/59)	14 (4.1)		0.1911	23 (6.8)	2.2 [1.210–3.852]	0.0136*	24 (7.1)	2.1 [1.146–3.634]	0.017*	11 (3.2)	2.3 [1.027–5.147]	0.0362*
	Without (95/280)	46 (13.6)			64 (18.9)			70 (20.6)			25 (7.4)		

*n* = 339 (breeder dogs was excluded from this analyze). CI: confidence interval. *p*-values: *, **, ***, **** significant difference at *p* < 0.05, *p* < 0.01, *p* < 0.001 and *p* < 0.0001, respectively.

Regarding anthelmintic treatment within the past 3 months, 23.4% (85/363) of dogs had been treated, 7.2% (26/363) had not received treatment, and in 69.4% (252/363) of cases, treatment status was unknown (Supplementary Table S5).

Puppies under 6 months of age were 4.5 times more likely to harbor protozoan infections, and the likelihood of monoparasitic infections was 3.5 times higher within this age group. Conversely, dogs aged 1–10 years showed a significantly reduced risk of protozoan infection (OR = 0.35; 95% CI, 0.1966–0.6237; *p* = 0.0006) and monoparasitism (OR = 0.44; 95% CI, 0.2502–0.7822; *p* = 0.0068). The presence of diarrhea was significantly associated with protozoan infections (OR = 2.2; 95% CI, 1.210–3.852; *p* = 0.0136), as well as with both monoparasitism (OR = 2.1; 95% CI, 1.146–3.634; *p* = 0.0170) and polyparasitism (OR = 2.3; 95% CI, 1.027–5.147; *p* = 0.0362). No statistically significant differences were observed between parasitism prevalence and sex (*p* = 0.4560) or breed (*p* = 0.1055) (Supplementary Table S7).

Regarding cat samples, the characteristics of all collected specimens (*n* = 345) are summarized in Table 7.

**Table 7:** Demographic and environmental characteristics of cats according to intestinal parasite status.

Variable	No. (%) positive samples	No. (%) negative samples	Total
Origin			
Veterinary clinics/hospitals (owned cats)	31 (9)	108 (31.3)	139 (40.3)
District of Aveiro	3 (0.9)	1 (0.3)	4 (1.2)
District of Coimbra	24 (6.9)	92 (26.7)	116 (33.6)
District of Lisboa	4 (1.2)	15 (4.3)	19 (5.5)
Municipal kennels (stray cats)	43 (12.5)	42 (12.2)	85 (24.6)
District of Aveiro	0 (0)	6 (1.7)	6 (1.7)
District of Coimbra	40 (11.6)	25 (7.2)	65 (18.8)
District of Guarda/ Viseu	3 (0.9)	11 (3.2)	14 (4.1)
CED program (stray cats)	73 (21.2)	48 (13.9)	121 (35.1)
District of Coimbra			
Age group			
<6 months	19 (5.5)	9 (2.6)	28 (8.1)
6–11 months	25 (7.2)	31 (9)	56 (16.2)
1–10 years	101 (29.3)	139 (40.3)	240 (69.6)
>10 years	2 (0.6)	19 (5.5)	21 (6.1)
Sex			
Female	80 (23.2)	110 (31.9)	190 (55.1)
Male	67 (19.4)	88 (25.5)	155 (44.9)
Breed			
Mixed breed	147 (4.6)	189 (54.8)	336 (97.4)
Purebred	0 (0)	9 (2.6)	9 (2.6)
Season			
Autumn	80 (23.2)	64 (18.5)	144 (41.7)
Winter	58 (16.8)	109 (31.6)	167 (48.4)
Spring	5 (1.4)	20 (5.8)	25 (7.2)
Summer	4 (1.2)	5 (1.4)	9 (2.6)
Diarrhea			
With	9 (2.6)	7 (2)	16 (4.6)
Without	138 (40)	191 (55.4)	329 (95.4)

Of these, 147 cats tested positive for intestinal parasites (42.6%), with 95 exhibiting infection by a single parasitic species (Table 8).

**Table 8:** Distribution of intestinal helminth and protozoan species in cats: prevalence of mono- and polyparasitism.

Parasites	No (%) (*n* = 211)	Monoparasitism (*n* = 95)	Polyparasitism (*n* = 116)
Helminths			
*Aelurostrongylus abstrusus*	22 (10.4)	5 (5.3)	17 (14.6)
Ancylostomatidae*	58 (27.5)	18 (19)	40 (34.5)
*Dipylidium caninum*	2 (0.9)	1 (1.05)	1 (0.9)
*Mesocestoides* spp.	1 (0.5)	1 (1.05)	0 (0)
Toxocara cati*	64 (30.3)	33 (34.7)	31 (26.7)
*Trichurida*	1 (0.5)	0	1 (0.9)
*Troglostrongylus*	1 (0.5)	0	1 (0.9)
Total of helminths species	149 (70.6)	58 (61.1)	91 (78.5)
Protozoa			
Giardia lamblia*	15 (7.2)	10 (10.5)	5 (4.3)
*Cystoisospora* spp.	38 (18)	22 (23.2)	16 (13.7)
*Cryptosporidium* spp.*	7 (3.3)	4 (4.2)	3 (2.6)
Toxoplasma gondii*	2 (0.9)	1 (1)	1 (0.9)
Total of protozoa species	62 (29.4)	37 (38.9)	25 (21.5)

*Zoonotic species.

A total of 11 different parasite species were identified in cats, encompassing intestinal nematodes (Ancylostomatidae, *T. cati*, and *Trichurida* eggs), respiratory nematodes (*Aelurostrongylus abstrusus* and *Troglostrongylus*), cestodes (*Dipylidium caninum* and *Mesocestoides* spp.), and protozoa, including *G. lamblia*, *Cystoisospora* spp., *Cryptosporidium* spp., and *Toxoplasma gondii*.

In contrast to the dog findings, helminths were more prevalent in cats (70.6%) than protozoa (29.4%). Among helminths, the roundworm *T. cati* (30.3%) and Ancylostomatidae (27.5%) were the most commonly detected.

Regarding anthelmintic treatment within the past 3 months, 18.0% (62/345) of cats were dewormed, 55.3% (191/345) were not, and the treatment status was unknown for 26.7% (92/345) (Supplementary Table S6).

Among the studied population, pet cats exhibited a significantly lower risk of parasitic infection (*p* < 0.0001), whereas stray cats were found to be 4.5 times more likely to be parasitized (Table 9). Regarding infection type and parasite diversity, stray cats demonstrated a 7.4-fold higher risk of helminth infections, were 10 times more likely to experience polyparasitism, and had twice the likelihood of monoparasitism compared to pet cats (Table 10). Age-related differences were notable; kittens younger than 6 months showed a significantly elevated risk of parasitism (*p* = 0.0085), while senior cats older than 10 years exhibited the lowest risk (OR = 0.13; *p* = 0.0011) (Table 9).

**Table 9:** Association between cat origin, demographic characteristics, and intestinal parasite prevalence.

Variable	Positive/ total samples	Prevalence (%)	OR (95%CI)	*р*-value
Origin				
Veterinary clinics/hospital (pet cats)	31/139	22.3	0.23 [0.1362–0.3643]	<0.0001****
Municipal kennel (stray cats)	43/85	50.6		0.1007
CNR program (stray cats)	73/121	60.3	3.1 [1.955–4.830]	<0.0001****
Total of stray cats	116/206	56.3	4.5 [2.745–7.343]	<0.0001****
Age group				
<6 months	19/28	67.9	3,2 [1.382–7.338]	0.0085*
6–11 months	25/56	44.6		0.769
1–10 years	101/240	42.1		0.813
>10 years	2/21	9.5	0.13 [0.0296–0.5126]	0.0011**
Breed				
Mixed breed	147/336	43.8	∞ [1.987 to ∞]	0.0118*
Purebred	0/9	0		
Season				
Autumn	80/144	55.5	2.5 [1.598–3.872]	<0.0001****
Winter	58/167	34.7	0.53 [0.3486–0.8211]	0.0047**
Spring	5/25	20	0.31 [0.1260–0.8107]	0.0202*
Summer	4/9	44.4		>0.999

CI: confidence interval. *p*-values: *, **, ***, **** significant difference at *p* < 0.05, *p* < 0.01, *p* < 0.001, and *p* < 0.0001, respectively.

**Table 10:** Association between cat origin and prevalence of intestinal parasites: odds ratios and statistical significance.

Variable	Category	Helminths			Protozoa			Monoparasitism			Polyparasitism		
		No. (%)	OR [95% CI]	*p-*value	*n* (%)	OR [95% CI]	*p-*value	*n* (%)	OR [95% CI]	*p-*value	*n* (%)	OR [95% CI]	*p-*value
Origin	Stray cats (116/206)	93 (27)	7.4 [4.025–13.210]	<0.0001****	38 (11)		0.379	68 (19.7)	2 [1.222–3.409]	0.0067**	48 (13.9)	10.3 [3.833–27.090]	<0.0001****
	Pet cats (31/139)	14 (4.1)			20 (5.8)			27 (7.8)			4 (1.2)		
Age group	<6 months (50/68)	12 (3.5)		0.1999	12 (3.5)	4.4 [1.899–9.443]	0.0006***	13 (3.8)	2.5 [1.153–5.343]	0.0266*	6 (1.7)		0.4046
	6–11 months (2/8)	23 (6.8)		0.0836	2 (0.6)	0.15 [0.036–0.594]	0.0028**	16 (4.6)		0.8707	9 (2.6)		0.8387
	1–10 years (98/281)	72 (21.2)		0.6130	42 (12.2)		0.6425	64 (18.6)		0.6019	37 (10.7)		0.8709
	>10 years (1/6)	0 (0)	0 [0–0.343]	0.0004***	2 (0.6)		0.5482	2 (0.6)		0.0754	0 (0)		0.0541

CI: confidence interval. *p*-values: *, **, ***, **** significant difference at *p* < 0.05, *p* < 0.01, *p* < 0.001, and *p* < 0.0001, respectively.

Furthermore, kittens were 4.4 times more likely to be infected with protozoa (*p* = 0.0006) and 2.5 times more likely to display monoparasitic infections (*p* = 0.03) (Table 10).

Seasonality was identified as a significant risk factor, with the highest parasite prevalence observed during autumn (55.5%), showing a strong statistical association (*p* < 0.0001). In contrast, no statistically significant differences were found for sex (*p* = 0.9129) or the presence of diarrhea (*p* = 0.3050) (Supplementary Table S8).

## 4 Discussion

In the present study, we aimed to estimate the prevalence of intestinal parasites in dogs and cats between November 2022 and April 2025, in Portugal. In addition, we evaluated selected epidemiological and clinical factors associated with these infections. Our results indicate that the overall prevalence of parasites in dogs was 41.6% and in cats was 42.6%, Over the past 15 years, several studies have investigated the prevalence of intestinal parasites in both domestic and stray populations across different regions of the country, including the northern region (15, 32, 37, 38), the Lisboa region (30), the southern region (26), and in the islands of Terceira and São Miguel in the Azores (40). These studies have consistently reported a high prevalence varying from 16 to 90.7% of parasitic infections in among dogs and cats, with higher prevalence’s infection rates observed for in stray and shelter animals.

The prevalence of parasite infection in dogs and cats reported here (41.6 and 42.6%, respectively) is similar to that reported in a previous study including pet and shelter animals (41.8%) (26), though lower than that reported for dogs from small-ruminant farms in northern Portugal (58.8%) (37). Variations in parasitic prevalence within the same country are not unexpected and may be attributed to differences in the investigated animal population, specific regional climates, number of samplings, anthelmintic protocols, diagnostic sensitivity, and combined use of different techniques.

Coprological methods and microscopic examination of fecal samples were employed to detect parasite eggs, oocysts, cysts, and larvae. Adult parasites and tapeworm segments (proglottids) were identified through macroscopic examination. To improve sensitivity for detecting protozoa, particularly *Giardia* spp., a zinc sulfate flotation solution with a specific gravity (10) was used, along with an immunochromatography test applied off-label for animal diagnostics. Coprological techniques remain a valuable tool for routine diagnostics and epidemiological surveys of parasitic infections. However, these methods have limited sensitivity, especially in cases of low-intensity infections or when parasites intermittently shed eggs, cysts, or oocysts. This restricts detection to only those fecal stages that are shed, thereby capturing only a fraction of the overall parasite prevalence (39). Regarding the sampling protocol, the study sought to include a diverse range of animal populations, namely pets, strays, and breeding animals, to ensure representation of the major subpopulations of dogs in Portugal. However, certain groups such as farm dogs, hunting dogs, and working dogs (e.g., police dogs) were underrepresented.

Of the four main parasites found in dogs, *G. lamblia* (44.7%), Ancylostomatidae (16.5%), *C. canis* (15.4%), and *T. canis* (14.9%) were prominent. Among these, only *C. canis* lacks recognized zoonotic potential. Furthermore, protozoa were the most frequently detected parasite group, similar to results reported for owned dogs in Serbia (19) and contrary to what observed in Central Italy (41). This finding is likely related to the combined use of different methods for parasite detection. In the present study, the combination of a flotation technique with a *Giardia* copro-immunochromatography test, increased diagnostic sensitivity. Additionally, the main anthelmintic compounds routinely used in deworming protocols for dogs in Portugal, such as praziquantel and macrocyclic lactones (avermectins and milbemycins), are ineffective against protozoan parasites (3, 34), suggesting that the selective pressure exerted by these anthelmintic drugs may contribute to an increased prevalence of protozoan infections.

In our study, *Giardia* spp. was identified as the most prevalent intestinal parasite in dogs, consistent with other European studies (22, 26, 42, 43). Notably, the observed prevalence of *Giardia* was 44.7%, higher than recently reported in other European countries, for example, 5% in kenneled dogs in central Italy (41), and 7–16% across various regions of Europe (20, 44–48). Even within Portugal, reported prevalence rate of *Giardia* infections have ranged from 7.4 to 33.8% (26, 38, 49–51).

Several factors may explain the high level of *Giardia* spp. detected in the present study. Indeed, the protozoan has a direct life cycle, and infection occurs through the ingestion of cysts shed by infected animals or humans, or indirectly via contaminated water or food. *Giardia* cysts are highly resistant and remain infective in environment for extended periods, while their short pre-patent period further facilitates rapid transmission and maintenance within host populations. Close contact among dogs within overcrowding and inadequate sanitary conditions in confined environments such as breeding facilities promote efficient transmission of *Giardia* spp. (52, 53). Moreover, urban parks represent important hotspots for *Giardia* transmission (7, 54, 55). As previously mentioned, another key factor contributing to the widespread dissemination of *Giardia* is that the main deworming protocols currently used are ineffective against protozoan parasites (3).

In the current study, breeder dogs showed the highest prevalence of *Giardia* infection (87.5%), with an 11.3-fold increased risk compared to stray and pet dogs. Caution is warranted as this refers to a single breeder facility housing Weimaraner and Border Collie dogs. Breeder animals are generally more susceptible to parasites due to factors such as higher population density and the vulnerability of puppies with underdeveloped immune systems. Additionally, maternal immunosuppression during pregnancy and postpartum may increase susceptibility to parasites such as *T. canis*, *Giardia* spp., and *Cystoisospora* spp. (42).

Pet dogs were the least susceptible group to harbor parasites (23.8%), with statistically significant differences when compared with other groups. Similar prevalence (20.8%) was reported in the Porto district (38). Given that many owned dogs receive proper care, have limited outdoor exposure, undergo regular anthelmintic treatment, and proper waste disposal, a lower prevalence (≤10%) would have been expected, similar to that reported in other European countries (17, 24, 56–58).

These findings highlight significant gaps in preventive measures that still need to be addressed. According to the European Scientific Counsel Companion Animal Parasites (ESCCAP) guidelines, deworming is recommended every 3 months (3). The data presented here indicate that 23.4% of the sampled population follow this recommendation. However, because treatment status was unknown for 69.4% of the sample, the observed adherence rate may not accurately reflect the true treatment practices in the studied animal population. A survey on Portuguese pet owners’ awareness and deworming practices found that only 33.7% administered internal deworming every 3 months (34).

Unsurprisingly, stray dogs exhibited a 2.3-fold higher risk of parasitism, with a 38.6% prevalence, exceeding recent reports (16%) (59). This discrepancy may be attributed to methodological differences: our study employed four distinct diagnostic techniques, whereas the comparison study relied solely on the mini-FLOTAC method. Increased parasite prevalence in stray and shelter dogs, compared to owned dogs, is due to unrestricted roaming and high density in shelters facilitating transmission. In Europe, stray dog parasitism ranges from 25 and 69% (14, 16). These results underscore the need for targeted surveillance and control strategies focusing on stray dogs, particularly upon shelter admission. Early intervention is essential to prevent the acquisition and spread of infections within the shelter environment, which can result from continuous exposure to infected animals or heavily contaminated surroundings.

Among geohelminths, hookworms (16.5%) and *T. canis* (14.9%) were most frequent, with helminth positivity significantly associated with stray dogs. Infection may originate prior to shelter admission or from exposure within the shelter. Morphological diagnosis of Ancylostomatidae eggs is challenging and species-level differentiation requires molecular methods. The main reported dog hookworm species are *Ancylostoma caninum* and *Ancylostoma braziliense*, both known for their high pathogenicity, often causing anemia and enteritis. These species also pose a zoonotic risk, causing “cutaneous *larva migrans*” in humans. *Uncinaria stenocephala* is more prevalent in temperate regions and generally considered less pathogenic. *Ancylostoma ceylanicum*, is found mainly in Asia and Australia and is capable of infecting both dogs and cats (60).

The coccidian *Cystoisospora canis* was detected at 15.4%, similar of reported in other European studies (<17%) (20, 23, 27, 38, 41, 61). Previous Portuguese studies reported prevalence rates <14%, with such higher values specifically observed in pet dogs with gastrointestinal signs (26, 32, 37–49, 59). Indeed, of our positive samples, 31.1% present abnormal consistency (score 4–7), suggesting intestinal disorders.

Concerning trichuroid parasites, in our study we detected *Trichuris vulpis*, at a prevalence of 4.3%. Reported values from Portugal varies from 1.1 to 49.5% (32, 37, 38). An association was observed between higher prevalence’s of this nematode and hunting dogs, in Portugal (32) and Spain (20). Despite having a direct life cycle that does not involve an intermediate host, the increased prevalence in hunting dogs may be linked to exposure to environments contaminated by feces of wild canids, which can also be parasitized and serve as potential reservoirs of infection.

Age under 6 months was a significant risk factor for parasitism, especially protozoan infection (4.5-fold). Furthermore, puppies showed significant association with monoparasitism (OR = 3.5; *p* < 0.0001), while dogs aged 1–10 years had reduced risk of protozoan infection and monoparasitism. These findings reflect early-life susceptibility to a predominant parasite, limited exposure to other species, and immature immune defenses, which gradually changes as animals age and environmental exposure increases. Maternal antibody protection wanes within the first weeks of life, creating a window of vulnerability before the pup’s own adaptive immunity becomes fully functional. Behavioral factors, such as increased oral exploration and frequent contact with contaminated environments, further elevate the risk of exposure to parasitic stages, such as helminth eggs, protozoan cysts, or larvae. Consequently, young dogs (puppies) constitute a risk group, in which infections often occur at higher prevalence rates and may result in severe clinical outcomes such as gastrointestinal disturbances, malabsorption, anemia, and impaired growth.

Diarrhea correlated significantly with parasitic positivity, particularly protozoa, and both mono- and polyparasitism. Parasite colonization disrupts mucosal integrity, leading to malabsorption and secretory diarrhea, often accompanied by mucus or blood. Clinical signs aid early diagnosis and management.

Sex and breed were not significant risk factors, consistent with previous studies (27, 29, 57).

In cats, three categories were studied: owned (40%), stray cats housed in Municipal kennels (25%), and Capture-Neuter-Return (CNT) program cats (35%). The CNT program, also known as Trap-Neuter-Return (TNR), is a humane approach to managing free-roaming cat populations while also collecting epidemiological data. As part of the program, stray cats are captured, sterilized, and subsequently returned to their original habitats (62). Stray and free-roaming cats exhibited 4.5-fold higher parasite prevalence, acting as environmental reservoirs. This finding aligns with our observations in dogs and is consistent with results reported in previous reports (16, 63).

Helminths were more prevalent (70.6%) than protozoa in cats, showing an opposite trend from dogs. Major parasites were *Toxocara cati* (30.3%), Ancylostomatidae (27.5%), *Cystoisospora* spp. (18%), and respiratory nematodes (11.4%). *Toxocara* spp. emerged as a major concern in our study, being the most prevalent parasite diagnosed in cats (30.3%) and the second more prevalent helminth in dogs (18.5%). This finding is of significant public health relevance, as *Toxocara* eggs are highly resistant in the environment (64). Toxocariasis is listed among the five most neglected parasitic infections according to the U. S. Centers for Disease Control and Prevention (65). Humans can acquire the infection through ingestion of infective *Toxocara* eggs, primarily via contact with contaminated soil. Such infections can result in extraintestinal pathologies, including visceral larva migrans, ocular larva migrans, covert toxocariasis, and neurotoxocariasis. Recent systematic review and meta-analysis demonstrated that contact with dogs and cats is a significant risk factor for human toxocariasis, particularly among children and in specific regions (America, Middle East and Western Pacific) (93). A study conducted in Portugal reported a seroprevalence of 18.8% for *Toxocara* among 846 inhabitants (6), similar of the reported global prevalence (19%) (94).

Respiratory nematodes may be often diagnosed by coprological methods, as they present transient intestinal stages. In our study, three different species were observed in cats: *Aelurostrongylus abstrusus* (10.4%), *Troglostrongylus brevior* (0.5%) and *Eucoleus aerophilus* (syn. *Capillaria aerophila*) (0.5%). Despite the use of a low-density solution, such as zinc sulfate solution, may occasionally allow the detection of larvae, their sensitivity is poor and often fails to detect infections due to insufficient sample size and the damaging effects of the flotation media (10). For that reason, the reported prevalence is likely underestimated, and veterinarians should give them particular attention, as important etiological agents of feline respiratory disease. By including these parasites in the differential diagnoses of cats presenting respiratory signs, especially in endemic areas, veterinarians can help reduce morbidity, improve animal welfare, and limit the spread of infection within feline populations.

The low prevalence of cestodes observed in cats (1.4%) and their absence in dogs may be underestimated due to several diagnostic limitations. These include the low sensitivity of macroscopic examination and flotation technique, since taeniid eggs are denser and less likely to float, as well the examination of only a single fecal sample per animal. Furthermore, the inconsistent shedding of proglottids and intermittent egg release, the presence of immature or non-gravid adult worms that have not yet produce eggs, and uneven distribution of cestode eggs in feces may all contribute to an underestimation of true prevalence (24, 66). Prevalence rates ranging from 0.51 to 1.7% have been reported in farm dogs of Northern Portugal (37), and 1.98% in hunting dogs (32). In contrast, a much higher value was recorded in Lisbon, where a prevalence of 75.3% was observed among stray cats (30). However, it’s important to point out that the latter study included necropsy in its diagnostic methodology, which greatly increase the sensibility for detecting cestodes infections compared to flotation techniques. Furthermore, the life cycle of most tapeworms, except the flea-borne *Dipylidium caninum* identified in this study, typically relies on ingestion of an infected intermediate host, either through predation or exposure to contaminated slaughterhouse waste (67). However, this conventional predator–prey transmission route is highly unlikely to occur in privately owned animals living in urban environments. In our study, the recovered tapeworms include *Dipylidium caninum*, at a lower prevalence of 0.9%. This value is lower than the 6% prevalence reported in stray dogs from Northern Portugal (Guimarães city) (15) and in client-owned dogs across Europe (68).

One of the most noteworthy findings of our study was the detection of *Mesocestoides* spp. eggs (0.5%). In Portugal, data on this parasite remain extremely scarce. To date, the only documented evidence consists of a recent conference poster reporting its detection in 2 out of 87 cats examined in Lisbon (69). Across Europe, *Mesocestoides* spp. infections occur sporadically in domestic cats, and as it requires the ingestion of an infected intermediate host, the parasite is more detected in regions where cats have outdoor access and engage in predation on small vertebrates, key factors that increase exposure risk. Clinical manifestations in cats may include gastrointestinal upset or, in cases where tetrathyridia proliferate in body cavities, more severe systemic disease. A global data review reported pooled prevalence of 8.32% in cats and 7.97% in dogs (70). In Europe, reported prevalence rates of *Mesocestoides* spp. infections range from <0.1 to13.8% (29, 67, 71–75). Despite its generally lower prevalence, this parasitosis can follow a very severe course if it progresses to peritoneal and pleural metacestodosis, as documented in a wild cat found deceased in Croatia (76).

Concerning intestinal protozoa, two *Cystoisospora* species were identified among the examined cats (overall prevalence of 18%): *C. felis* (17.1%) and *C. rivolta* (0.9%). In a previous study, a much higher prevalence of *C. rivolta* (46.3%) was reported in stray cats from Lisbon (30). Of particular note is *Giardia,* which was identified as the second most frequently detected protozoa (7.1%) and the most prevalent in dogs. *Giardia* spp. causes gastrointestinal illness (giardiosis) and may manifest as acute, chronic, or asymptomatic infection. Common clinical signs include diarrhea, vomiting, abdominal pain, and flatulence. With notable zoonotic potential, several assemblages can infect both humans and animals. Dogs and cats may be affected by zoonotic assemblages A or B and by dog-specific assemblages C or D, and cat-specific assemblage F, respectively (77). Although human giardiasis is linked to assemblages A and B, occasional infections involving domestic animal-adapted assemblages have been documented, confirming zoonotic transmission, while rare, is possible (78–80). Analysis of canine and feline samples from Portugal demonstrated a predominance of pet-specific assemblages (26, 50, 51). In contrast, a previous investigation of 31 canine fecal samples identified 68% as zoonotic assemblage A and only 13% as dog-specific assemblages C and D (81), underscoring the potential role of these animals in *Giardia* transmission to humans. Monitoring and controlling *Giardia* infections in companion animals is crucial to mitigate zoonotic transmission, with molecular characterization essential for determining genotypes.

Additionally, *Toxoplasma gondii* oocysts were detected in 0.9% of the samples, however, this result requires further confirmation through molecular assays (83, 84).

To improve the detection of *Cryptosporidium* spp., a differential staining - Modified Ziehl–Neelsen were applied. *Cryptosporidium* spp. represents an important cause of enteric infection in dogs and cats, with infections ranging from subclinical carriage to acute or chronic diarrhea. Given their zoonotic potential and the difficulty of distinguishing cryptosporidiosis from other gastrointestinal diseases based solely on clinical signs, reliable diagnostic testing is essential to ensure accurate case identification, guide treatment decisions, and limit environmental dissemination of oocysts. Prevalence’s obtained in the present study were similar (3%), for dogs and cats. Across Europe, reported prevalence rates range from 0.6 to 11.9% in dogs (7, 22, 46, 63, 85, 86) and from 4.4 to 8.8% in cats (63, 86).

Although several parasitic species have previously been documented in Portugal, *Sarcocystis* sp., *Ollulanus tricuspis*, *Taenia taeniformis*, *Joyeuxiella pasqualei* and *Diploplylidium nölleri* in cats (30), as well *Capillaria* (15, 37), *Spirometra* (37), *Taeniidae* (32, 37), *Echinococcus granulosus* (87), *Sarcocystis* sp. (7) and *Angiostrongylus vasorum* (40) in dogs, none of these parasites were detected in the present study, perhaps due to geographic and/or methodological factors.

Our data showed seasonal prevalence peaked in autumn and spring among dogs, and in autumn for cats, periods typically characterized by moderate temperatures and variable precipitation.

Portugal exhibits a temperate Mediterranean climate with regional variability, which could play an important role in parasite transmission dynamics. In central and northern districts such as Coimbra, Aveiro, and Viseu, winters are mild and relatively wet, with average temperatures ranging from approximately 6–15 °C and monthly rainfall often exceeding 90–150 mm, while summers are warm and dry, with temperatures reaching 20–30 °C and minimal precipitation (88–91). Coastal areas like Lisbon, experience milder temperatures (11–30 °C annually) and moderate rainfall, whereas southern inland regions such as Évora are characterized by hotter and drier conditions, with summer temperatures frequently exceeding 30 °C and very limited rainfall (92, 93). These environmental conditions favor the survival and development of many intestinal parasite stages during the cooler and wetter months, enhancing environmental contamination and transmission risk (39). In contrast, the hot and dry summer period may reduce the viability of some parasites, although resistant forms can persist, allowing continued low-level transmission. Overall, this climatic pattern supports seasonal fluctuations in parasite dissemination, with increased risk during periods of higher humidity and moderate temperatures. Interpretation of seasonal trends should be made with caution, as sampling was not evenly distributed across seasons. In particular, winter samples were overrepresented, while relatively few samples were collected during spring and summer. Despite this imbalance, statistically significant seasonal associations were still observed, suggesting that the detected effects are unlikely to be driven solely by sampling bias. Nevertheless, future studies with more balanced seasonal sampling are warranted to confirm these findings.

Our findings highlight that stray and shelter animals harbor significantly higher intestinal parasite burdens compared to household pets. Stray animal populations play a significant role in environmental contamination and the amplification of zoonotic risk, particularly endangering vulnerable populations such as children and immunocompromised individuals. Free-roaming dogs and cats often lack regular veterinary care, including routine deworming, which allows intestinal parasites to persist and be continuously shed into the environment through feces. This leads to the widespread contamination of soil, water, and public spaces such as parks and playgrounds with infective stages, including parasite eggs, larvae, and protozoan cysts. Strengthening preventive strategies may help reduce environmental contamination and limit the risk of zoonotic transmission.

The strong associations observed between parasite positivity and factors such as origin, age, and abnormal fecal consistency emphasize the multifactorial nature of parasitic infections in companion animals. Addressing these risks requires targeted surveillance, improved preventive healthcare, and public awareness campaigns to mitigate parasite transmission in both animal and human populations.

Moreover, this study underscores the critical need for ongoing molecular characterization of *Giardia* and *Cryptosporidium* genotypes circulating among canine and feline populations. Understanding the zoonotic potential of specific genotypes will enhance risk assessment and inform the development of more effective diagnostic, therapeutic, and control strategies. Future research should focus on integrating molecular epidemiology with traditional parasitology to offer comprehensive insights into parasite transmission dynamics and to support the implementation of evidence-based interventions aiming to safeguard both animal and public health.

## 5 Conclusion

In conclusion, this study provides a comprehensive assessment of intestinal parasite prevalence and associated risk factors in dogs and cats across diverse populations in Portugal. The markedly higher parasite burdens in stray and shelter animals compared to pets underscore their critical role as reservoirs sustaining environmental contamination and zoonotic transmission cycles. Key host factors such as origin, age, and fecal consistency abnormalities were significantly linked to parasite positivity, reinforcing the need for targeted surveillance and tailored interventions to reduce infection rates in at-risk groups. Furthermore, the findings highlight the importance of integrating molecular diagnostic techniques to characterize zoonotic *Giardia* and *Cryptosporidium* genotypes circulating in companion animals. Such molecular insights will facilitate precise epidemiological tracking and inform more effective control strategies, ultimately benefiting animal health and mitigating public health threats. Concerted efforts encompassing routine deworming, responsible pet ownership, stray animal management, and public health education remain essential to curtail the burden of intestinal parasitism in both animals and humans.

## Data Availability

The original contributions presented in the study are included in the article/Supplementary material, further inquiries can be directed to the corresponding author/s.
