# Supplementary material for: Beyond the clinical eye: mapping intestinal parasitic infections and its risk factors among dogs and cats across Portugal
Source: Front Vet Sci. 2026 Jul 1;13:1814054. doi: 10.3389/fvets.2026.1814054 (PMC13371433; doi:10.3389/fvets.2026.1814054)
Supplement: Supplementary file 1 [file Data_sheet_1.pdf]

Supplementary Material

Table S1. Monthly climate variables (October–December) for Portugal, in 2022. Data extracted from the Instituto Português do Mar e da Atmosfera (IPMA) website, https://www.ipma.pt/pt/index.html.

**2022**		OCT	NOV	DEC
Average Minimum Temperature (°C)	Aveiro	14,8	11,4	12,2
	Coimbra	14,2	10,4	11,2
	Évora	14,1	9	9,6
	Guarda	11,6	6	5,7
	Lisboa	16,4	12,2	12,1
	Viseu	12,9	7,8	7,7
Average Maximum Temperature (°C)	Aveiro	22,2	18,3	17,3
	Coimbra	23,8	17,2	16
	Évora	26,5	18,7	16,9
	Guarda	18,5	11,5	9
	Lisboa	24,8	18,7	17,2
	Viseu	21,3	14,3	12,2
Total Precipitation (mm)	Aveiro	145	253,8	291,2
	Coimbra	116,1	198	219,9
	Évora	75,3	46,7	186,9
	Guarda	180,6	144,4	331
	Lisboa	102,6	89	368,4
	Viseu	163,2	272,2	320,1

OCT: October; NOV: November; DEC: December.

Table S2. Monthly climate variables (January–December) for Portugal, in 2023. Data extracted from the Instituto Português do Mar e da Atmosfera (IPMA) website, https://www.ipma.pt/pt/index.html.

**2023**		JAN	FEB	MAR	APR	MAY	JUN	JUL	AUG	SEP	OCT	NOV	DEZ
Average Minimum Temperature (°C)	Aveiro	7,6	7	10,4	12,1	14,5	17,3	16,6	17,4	16.6	15,7	12,4	7,6
	Coimbra	6,2	7	9,3	11,1	12,8	15,7	15	16,5	15.8	15,1	11,4	7,2
	Évora	4,4	4,3	7,3	9,8	12,2	16,4	15,9	17,6	15.6	14,1	9,3	5,3
	Guarda	1,2	1,3	5,2	8	8,2	13,5	13,3	15,2	13	-	6,4	2,4
	Lisboa	8,2	7,5	10,5	13,1	15	17,4	17,5	19	17.4	16,2	12,5	8,7
	Viseu	3,4	4	6,7	9	10,1	14,3	13,2	15	14	14	8,4	4,8
Average Maximum Temperature (°C)	Aveiro	15,1	16,7	18,3	21,3	22,6	24,3	24	25,4	25	24	18,4	14,8
	Coimbra	13,5	16	18,6	23	23,9	26,4	27,4	30,2	26.1	24	17,4	14,1
	Évora	14,6	16,5	20,7	27,2	27,3	32,2	34,3	36	28.9	26,2	19	15
	Guarda	7,5	9,2	13,6	18,5	19	23,3	26,4	28,5	20.9	18,4	12,8	9,3
	Lisboa	14,8	16,3	19,6	25	25,4	28,7	29,2	31,7	27.2	25,2	18,9	15,3
	Viseu	10,7	13,1	15,9	20,9	21,7	25,6	27,6	30,5	24	21,2	15	11,7
Total Precipitation (mm)	Aveiro	149	1,8	37,2	18,7	14,6	31,7	6,2	0,3	78.9	249,7	183,3	83,5
	Coimbra	164,6	3,6	72,6	21,6	104,5	47,2	7,3	3,1	72.3	277,7	164,9	70,2
	Évora	41,2	2,2	22,6	4,4	12,2	16	0,3	0	39.2	146,8	63,3	20
	Guarda	100,8	4,6	55,2	13,7	36,9	93,7	0,2	0,1	159.1	253,2	105,1	48,2
	Lisboa	61,1	2,2	25,4	9,8	16,6	28,4	0	0,1	68.6	131,6	102,4	36,6
	Viseu	182,3	2,8	67	21,5	58,7	84,3	2,8	1,1	114.8	340	216	81,6

JAN: January; FEB: February; MAR: March; APR: April; MAY: May; JUN: June; JUL: July; AUG: August; SEP: September; OCT: October; NOV: November; DEC: December; - Values were not available.

Table S3. Monthly climate variables (January–December) for Portugal, in 2024. Data extracted from the Instituto Português do Mar e da Atmosfera (IPMA) website, https://www.ipma.pt/pt/index.html.

**2024**		JAN	FEB	MAR	APR	MAY	JUN	JUL	AUG	SEP	OCT	NOV	DEZ
Average Minimum Temperature (°C)	Aveiro	-	10	9,6	11,6	12,6	15,6	16,5	16,3	14,4	15,3	13,5	7,7
	Coimbra	8,7	9,5	9,1	10,8	11,2	14,2	15,5	15,5	13,9	14,3	12,9	7,3
	Évora	7	7,3	7,6	9	9,9	14	16	17,1	14,2	13,2	-	-
	Guarda	4,2	5	4,1	6,4	7,6	10,8	14,9	15,7	10,4	9,6	7,8	2,4
	Lisboa	9,6	11	10,6	12,9	13,4	15,9	17,8	18,5	16,7	16	13,3	8,6
	Viseu	6,5	7	6,1	8,4	9,1	12,4	15	15,5	12,1	11,5	10,1	4,6
Average Maximum Temperature (°C)	Aveiro	-	17,4	17,8	20,6	20,1	22,5	24,6	24,2	23	22,1	20,4	16,4
	Coimbra	15,9	17,4	17,3	22,1	22	24,8	28,2	28,7	25,6	21,6	19,5	15
	Évora	16,5	18,2	18,4	23	26,4	29,4	35,4	36,2	29,8	23,5	-	-
	Guarda	10,2	11,6	11	16,1	17,5	22,3	28,1	29,4	21,2	15,6	13	8,8
	Lisboa	16,4	18	18,1	22,3	23,4	25,2	29,8	30,1	26,8	22,9	20	15,7
	Viseu	13,1	14,5	14,2	19,7	19,7	23,7	29,1	31,2	24,1	18,4	16,7	12,8
Total Precipitation (mm)	Aveiro	117,2	135,6	187,7	63,3	66,2	33,3	22,2	0,9	49,4	168,8	52,9	8,5
	Coimbra	134,9	111,6	199,6	48,9	43,9	93,5	21,2	3,4	50,5	200,7	72,6	15,2
	Évora	111,9	52,7	146,8	27,3	3	13,6	9,8	0	5,4	130,6	68,3	2,2
	Guarda	159,1	90,2	198,7	49,7	29,8	82,5	20,9	0	24,3	215,6	116,2	5,3
	Lisboa	119,9	81,2	177,4	18,7	2,4	26,7	2,2	0,1	10,8	79	50,1	13,6
	Viseu	175,3	171,9	297,2	61,4	66,9	67,9	7,2	1,1	42,8	270,9	96,8	24,5

JAN: January; FEB: February; MAR: March; APR: April; MAY: May; JUN: June; JUL: July; AUG: August; SEP: September; OCT: October; NOV: November; DEC: December; - Values were not available.

Table S4. Monthly climate variables (January–April) for Portugal, in 2025. Data extracted from the Instituto Português do Mar e da Atmosfera (IPMA) website, https://www.ipma.pt/pt/index.html.

**2025**		JAN	FEB	MAR	APR
Average Minimum Temperature (°C)	Aveiro	9,1	9,2	9,5	11,8
	Coimbra	8,6	8,5	8,9	11
	Évora	6	6	7,4	9,6
	Guarda	3	3,7	3,1	6,3
	Lisboa	9,6	9,6	9,9	12,4
	Viseu	5,5	5,5	5,1	8,2
Average Maximum Temperature (°C)	Aveiro	16	16,7	17	19,9
	Coimbra	15	16,1	16,6	20
	Évora	15,5	17	17,3	20,9
	Guarda	8,7	10	9,7	14,3
	Lisboa	15,9	16,8	17	20,5
	Viseu	12	13,3	13,5	17,7
Total Precipitation (mm)	Aveiro	177,4	65	159,2	143,7
	Coimbra	164,1	50,4	175,5	148,2
	Évora	139,4	65,1	223,4	100,3
	Guarda	298,1	56,1	257	156,3
	Lisboa	153,1	134,7	192	125,4
	Viseu	283,9	47,8	217,7	134,9

JAN: January; FEB: February; MAR: March; APR: April.

Table S5. Dog sampling characterization: epidemiological and clinical variables.

**Characteristics**	**No. (%)**
**Origin**	
Veterinary clinics/hospital	63 (17)
Coimbra	34 (9)
Lisboa	29 (8)
Breeder kennel	
Évora	24 (7)
Municipal kennel	236 (65)
Aveiro	6 (2)
Coimbra	88 (24)
Viseu/Guarda	142 (39)
Private Dog Refuge	
Coimbra (Figueira da Foz)	40 (11)
**Age group**	
<6 months	68 (19)
6-11 months	8 (2)
1-10 years	281 (77)
>10 years	6 (2)
**Sex**	
Female	185 (51)
Male	178 (49)
**Breed**	
Mixed breed	307 (85)
Purebred	56 (15)
Beagle	2 (3.6)
Belgian Shepherd	1 (1.8)
Bernese Mountain	3 (5.3)
Border collie	7 (12.5)
Bulldog	1 (1.8)
French Bulldog	7 (12.5)
Labradoodle	1 (1.8)
Labrador Retriever	3 (5.3)
Miniature Schnauzer	1 (1.8)
Pinscher	3 (5.3)
Poodle toy	1 (1.8)
Portuguese pointer	1 (1.8)
Pug	1 (1.8)
Pug starling	1 (1.8)
Rough Collie	1 (1.8)
Samoyed	1 (1.8)
Teckel	2 (3.6)
Weimaraner	18 (32.1)
Yorkshire terrier	1 (1.8)
**Anthelmintic treatment lasting fewer than 3 months**	
Yes	85 (23.4)
Monotherapy	36 (9.9)
Isoquinoline-Pyrazines (Praziquantel)	31 (8.5)
Macrocyclic Lactones (Avermectins and Milbemycins)	4 (1.1)
Nitroimidazole (Metronidazol)	1 (0.3)
Combination Therapy	49 (13.5)
Benzimidazoles (Fenbendazole) + Praziquantel	2 (0.5)
Milbemycin oxime + Praziquantel	8 (2.2)
Milbemycin oxime + Praziquantel + Selamectin	1 (0.3)
Praziquantel + Tetrahydropyrimidines (Pyrantel embonate) + Pro-benzimidazole (Febantel)	15 (4.1)

Table S5.*(cont.)*

Pyrantel embonate + Febantel	23 (6.3)
No	26 (7.2)
Not known	252 (69.4)
**Season of fecal sampling**	
Autumn	56 (15)
Winter	255 (70)
Spring	46 (13)
Summer	6 (2)
**Fecal score**[a]	
1	4 (1.1)
2 -3 (normal)	246 (67.8)
4-5	47 (12.9)
6	65 (17.9)
7	1 (0,3)

[a] Fecal consistency according to the Purina stool chart

Table S6. Cat sampling characterization: epidemiological and clinical variables.

**Characteristics**	**No. (%)**
**Origin**	
Veterinary clinics/hospital	139 (40)
District of Aveiro	4 (1)
District of Coimbra	116 (34)
District of Lisboa	19 (5)
Municipal kennel	85 (25)
District of Aveiro	6 (2)
District of Coimbra	65 (19)
District of Guarda/Viseu	14 (4)
CED program	
District of Coimbra	121 (35)
**Age group**	
<6 months	28 (8)
6-11 months	56 (16)
1-10 years	240 (70)
>10 years	21 (6)
**Sex**	
Female	190 (55)
Male	155 (45)
**Breed**	
Mixed breed	336 (97)
Purebred	9 (3)
British Shorthair	3 (33.3)
Persian	1 (11.1)
Scotitsh Fold	2 (22.2)
Siamese	3 (33.3)
**Anthelmintic treatment lasting fewer than 3 months**	
Yes	62 (18)
Monotherapy	45 (13)
Benzimidazoles (Fenbendazole)	3 (0.9)
Macrocyclic Lactones (Avermectins and Milbemycins)	41 (11.9)
Nitroimidazole (Metronidazol)	1 (0.3)
Combination Therapy	17 (4.9)
Benzimidazoles (Fenbendazole) + Praziquantel	1 (0.3)
Milbemycin oxime + Praziquantel	10 (2.8)
Praziquantel + Tetrahydropyrimidines (Pyrantel embonate)	2 (0.6)
Praziquantel + Pyrantel embonate + Metronidazol	1 (0.3)
Praziquantel + Depsipeptide (Emodepside)	1 (0.3)
Praziquantel + Avermectin (Eprinomectin)	2 (0.6)
No	191 (55)
Not known	92 (27)
**Season of fecal sampling**	
Autumn	144 (42)
Winter	167 (48)
Spring	25 (7)
Summer	9 (3)
**Fecal score**[a]	
1	5 (1.5)
2-3 (normal)	296 (85.8)
4	26 (7.5)
5	2 (0.6)
6	15 (4.3)
7	1 (0.3)

Table S7. Statistical analysis for dogs.

Variable	Positive/ total samples	Prevalence	[95%CI]	*p* value
**Origin**				
**Veterinary clinics/hospitals** (Owned dogs)	15/63	23.8%	0.4 [0.1966-0.7098]	0.0018**
District of Coimbra	7/34	20.6%		
District of Lisboa	8/29	27.6%		
**Breeder kennel** (Owned dogs)				
District of Évora	21/24	87.5%	11.3 [3.379-36.19]	<0.0001****
**Municipal kennels** (Stray dogs)	91/236	38.6%	0.7 [0.4483-1.096]	0.1188
District of Aveiro	4/6	66.6%		0.2085
District of Coimbra	38/88	43.2%		0.2717
District of Guarda/Viseu	49/142	34.5%		
**Private dog refuge** (Stray dogs)	24/40	60%	2.3 [1.167-4.431]	0.0166*
**Age group**				
<6 months	50/71	70.4%	4.5 [2.6-7.7]	<0.0001
6-11 months	2/8	25%		0.4772
1-10 years	98/278	35.2%	0.3 [0.1760-04830]	<0.0001****
>10 years	1/6	16.7%		0.4072
**Sex**				0.456
Female	73/185	39.5%		
Male	78/178	43.8%		
**Breed**				0.1055
Mixed breed	122/307	39.7%		
Purebred	29/56	51.8%		
**Season**				
Autumn	30/56	53.6%		0.0556
Winter	96/255	37.6%	0.58 [0.3668-0.9189]	0.0203*
Spring	23/46	50%		0.2626
Summer	2/6	33.3%		>0.9999
**Diarrhea**				
Presence	42/66	63.6%	3 [1.1760-5.258]	<0.0001****
Absence	109/297	36.7%		

CI: confidence interval
p-values: *, **, ***, **** significant difference at $p<0.05$, $p<0.01$, $p<0.001$ and $p<0.0001$, respectively

Table S8. Associations of intestinal parasites presents in cats.

**Variable**	**Positive/ total samples**	**Prevalence**	**OR [95%Cl]**	***p* value**
**Origin**				
**Veterinary clinics/hospital** (owned cats)	31/139	22.3%	0.23 [0.1362-0.3643	<0.0001****
District of Aveiro	3/4	75%		
District of Coimbra	24/116	20.7%		
District of Lisboa	4/19	21.1%		
**Municipal kennel** (stray cats)	43/85	50.6%		0.1007
District of Aveiro	0/6	0%		
District of Coimbra	40/65	61.5%		
District of Guarda/Viseu	3/14	21.4%		
**CED program**m (stray cats)				
District of Coimbra	73/121	60.3%	3.1 [1.96-4.83]	<0.0001****
**Stray cats**	116/206	56.3%	4.5 [2.745-7.343]	<0.0001****
**Age group**				
<6 months	19/28	67.9%	3,2 [1.382-7.338]	0.0085*
6-11 months	25/56	44.6%		0.769
1-10 years	101/240	42.1%		0.813
>10 years	2/21	9.5%	0.13 [0.0296-0.5126]	0.0011**
**Sex**				0.9129
Female	80/190	42.1%		
Male	67/155	43.2%		
**Breed**				
Mixed breed	147/336	43.8%		
Purebred	0/9	0%		
**Season**				
Autumn	80/144	55.5	2.5 [1.598-3.872]	<0.0001****
Winter	58/167	34.7	0.53 [0.3486-0.8211]	0.0047**
Spring	5/25	20	0.31 [0.1260-0.8107]	0.0202*
Summer	4/9	44.4		>0.999
**Diarrhea**				0.305
Presence	9/16	56.3%		
Absence	138/329	41.9%		

CI: confidence interval
p-values: *, **, ***, **** significant difference at $p<0.05$, $p<0.01$, $p<0.001$ and $p<0.0001$, respectively
